# Dr. Savage on the Relation between Post-Mortem Appearances and Pathological Processes

**Published:** 1899-12-16

**Authors:** 


### DR. SAVAGE ON THE RELATION BETWEEN POST-MORTEM APPEARANCES AND PATHOLOGICAL PROCESSES.

Granting, however, that certain groups of nerve diseases end by producing degenerative changes which are so similar as to suggest an identity of origin, a conclusion to which we are driven by the patient observations of microscopical investigations, we must not omit to take note of what Dr. Savage said at the same meeting on the relation between pathological processes and post-mortem appearances, because they are applicable to researches which are being carried out in many directions, and give a warning as to the interpretation of post-mortem observations which should not be lost sight of. Speaking of the relation between tabes and general paralysis of the insane, he said that he found it difficult to consider pathology only from the microscopical changes found in tissues after death, and although he at once allowed that in both general paralysis and in tabes they had similar evidences that destructive changes had been going on, yet he could not allow that as a consequence of finding such similar results one must therefore necessarily admit that exactly similar causes must have been at work in their production. The débris in a wrecked house, he said, was the same whatever might have been the cause of the conflagration by which it had been destroyed. Again, as showing that it is not always safe to deduce the processes which had been gone through from the results discovered, one had to acknowledge, from evidence on the clinical side, that as a rule toxic agents which were easily absorbed affected similar parts and tissues in similar ways although the agents themselves might be very different. The way in which such toxic agents acted was that they affected the least stable and the most developed tissues. Thus, taking the separate symptoms which occurred in general paralysis of the insane, they saw them all in the various stages of alcoholic intoxication; and with lead, even with influenza, they saw similar symptoms. The same parts were more or less affected by these different poisons. In regard to the particular point under discussion he said that he was not convinced that general paralysis of the insane and ataxia were the same, though he admitted that they probably had similar causes, and certainly left similar pathological débris. He was inclined to agree that decay of the neuron followed by secondary inflammation were parts of the diseased process in both, and that a toxic cause was the chief cause of this decay. As to their relation to syphilis, he said that the syphilitic was the most common toxin, though he believed that there might be an autotoxin in some cases resulting from rapid brain change, such as occurred in acute delirious mania.

